# Non-hormone replacement therapy to overcome premature ovarian insufficiency: advances in natural products and stem cells targeting autophagy

**DOI:** 10.3389/fendo.2025.1571021

**Published:** 2025-05-30

**Authors:** Xinxin Yang, Zhicheng Jia, Mengyu Shi, Yongqian Li, Guangheng Zhang, Peixuan Wang, Xinwei Sun, Wenlong Qi, Ying Guo

**Affiliations:** ^1^ The First Clinical Medical College, Shandong University of Traditional Chinese Medicine, Jinan, China; ^2^ Department of Gynecology, Guang'anmen Hospital, China Academy of Chinese Medical Sciences, Beijing, China; ^3^ Department of Reproduction and Genetics, Affiliated Hospital of Shandong University of Traditional Chinese Medicine, Jinan, China

**Keywords:** premature ovarian insufficiency, autophagy, molecular mechanisms, nonhormonal therapy, natural products, stem cells

## Abstract

Premature ovarian insufficiency (POI) is the most common cause of female infertility. With the increase in people’s bad life habits, the causative factors of POI have increased, and its incidence has shown a rising trend year by year. At present, the commonly used clinical treatment for POI is hormonal replacement therapy (HRT), but it is not universally applicable and is prone to cause subsequent complications, posing certain health risks to patients with POI. Therefore, exploring greener, safer, and more efficacious non-hormonal treatments can help to address the clinical challenges of POI-induced infertility better. Studies have shown that autophagy plays a key role in the development and degeneration of oocytes from their origin to the follicle and that any alteration in autophagy affects the ovarian reserve in the follicle. Moreover, certain natural products and human stem cells from different sources can treat POI by modulating the autophagic pathway and have shown good efficacy. Therefore, our study aimed to review and analyze the previous research-based literature on natural product and stem cell therapy based on the autophagy mechanism of POI, and provide new insights and references for related scholars to continue to explore the autophagy mechanism of POI and non-hormone-targeted therapeutic strategies in depth.

## 1 Introduction

Premature ovarian insufficiency (POI) is a pathological ovarian aging induced by multiple factors (1), characterized by primary or secondary amenorrhea of more than 4 months before the age of 40 in females, and follicle stimulating hormone (FSH) levels greater than 25 IU/L in 2 consecutive samples (2), its incidence rate before the age of 40 in women is 1% (3). POI has become one of the leading causes of infertility in women of childbearing age. Not only that, but it is also associated with a variety of health risks including psychological disorders, osteoporosis, cardiovascular diseases, autoimmune diseases, and even leads to an increased risk of death in patients. Presently, about 25% of POIs can be categorized as medical, with chemotherapy or radiotherapy, etc., being the main cause of POIs (4). In addition, with the increase in non-pharmacologic intervention triggers brought about by social development, such as smoking, drinking, staying up late and other unhealthy lifestyles, overexposure to plastic toxins, etc. (5), the incidence of POI has been increasing year by year. HRT and IVF are currently the most common and effective clinical treatments for POI. HRT refers to the use of hormones (primarily estrogen) to simulate estrogen levels in women of childbearing age with normal ovarian function (6), and this therapy has been shown to lead to an increased risk of diseases such as coronary heart disease, endometrial cancer, and breast cancer in women with POI (7) and not all patients with POI are suitable for HRT regimens, e.g., women with POI who have poor uterine conditions (endometrial thickness and poor angiogenesis) show resistance to HRT regimens (8). In addition, despite significant advances in IVF technology, its success rates depend on a variety of factors, including age, ovarian reserve, and oocyte quality, with a success rate of only about 37% per IVF cycle, which continues to decline with age due to the poor prognosis for women with reduced ovarian responsiveness (9). So the search for greener, safer, and more effective therapies is expected to provide new ideas for breakthroughs in the clinical management of POI. Natural products have received increasing attention in the biomedical field due to their multi-component, multi-target, and multi-pathway advantages, showing better efficacy in alleviating aging and resisting oxidative stress damage (10) without obvious toxic side effects (11). Moreover, stem cells have continuous self-renewal and differentiation functions, which can promote tissue repair and regeneration, and produce long-lasting therapeutic effects on damaged senescent cells and tissues, in particular, mesenchymal stem cells have unique advantages over other types of stem cells (12), which have been widely and intensively researched in the treatment of POI. In contrast to the frequently employed hormone replacement therapy, both of these treatment modalities exhibit low rejection rates and broad applicability. It is anticipated that they can decrease the incidence of various complications and enhance the clinical outcomes for patients suffering from POI.

The etiologies contributing to POI are complex and diverse (13), and approximately 90% of spontaneous Premature ovarian insufficiency has no clear underlying etiology (14, 15), and there is still an ongoing quest to understand the exact mechanisms underlying the development of POI. Autophagy, a process of self-degradation of intracellular components that maintain cellular and energetic homeostasis, was discovered as early as 1962 and is highly conserved among species (16). Research evidence suggests that autophagy plays an important role in sustaining and regulating ovarian primordial follicle reserve, anti-ovarian aging, and granulosa cell differentiation (17, 18). The disruption of autophagic mechanisms or excessive autophagic fluxes may lead to cell death, altering the quality and quantity of oocytes, and ultimately affecting female reproductive health which leads to POI (19, 20). The exploration of POI autophagy mechanisms and the discovery of targeting strategies are of great significance for protecting female fertility in reproductive age, improving the fertility level of fertile couples, and maintaining family and social harmony.

In this article, we retrospectively summarized the studies on the potential mechanisms of the autophagy pathway in regulating POI, as well as the current status of natural products and stem cells targeting autophagy in POI, to inform the research on the progress of therapeutic strategies for POI.

## 2 Autophagy overview

Autophagy is a major intracellular degradation pathway that exists in three main forms, namely: macroautophagy, chaperone-mediated autophagy, and microautophagy, and all three autophagic degradation pathways are centered on the lysosome (21), of which macroautophagy is the only most prevalent autophagic process that contains a double membrane autophagosome structure (22). In this article, we focused on macroautophagy, hereinafter referred to as “autophagy”. Autophagy is an intracellular degradation-recycling pathway with a dual effect (23) that encapsulates intracellularly damaged lipids, proteins, and organelles in double-membrane vesicles and transports them to lysosomes for fusion to form an autophagy-lysosome complex, which is then degraded into amino acids and small molecules and recycled to rejoin the intracellular biogenesis and plays a role in quality control and thus maintaining cellular homeostasis (24).

Autophagy mainly consists of several key steps: autophagy induction, autophagosome assembly and formation (including nucleation of autophagy-associated protein ATG at PAS, elongation of the separating membrane and maturation of autophagosomes, and transport of mature autophagosomes), fusion of autophagosomes and lysosomes, and degradation and reuse of autophagic lysosomal complexes, and the numerous autophagy-associated proteins ATGs and their core complexes endow the autophagy pathway with multiple activities that regulate and control the various stages of the autophagic process described above (25). These proteins and their core complexes can be divided into the following functional units: (1) ULK kinase core complex, including ULK1/2, ATG13, RB1CC1/FIP200, and ATG101; (2) Autophagy-specific class III phosphatidylinositol 3-kinase (PI3K) complex, including VPS34, VPS15, Beclin1, and ATG14L; (3) ATG9A transporter system, including ATG9A, WIPI1/2, and ATG2; (4) ATG12 ubiquitin-like ligand system, including ATG12, ATG7, ATG10, ATG5, and ATG16L1; (5) LC3/Atg8 ubiquitin-like ligand system, including LC3A/B/C, ATG7, ATG3, the above functional units are involved in the hierarchical regulation of autophagy, and form a tight cascade of reactions during autophagy.

**Figure 1:**
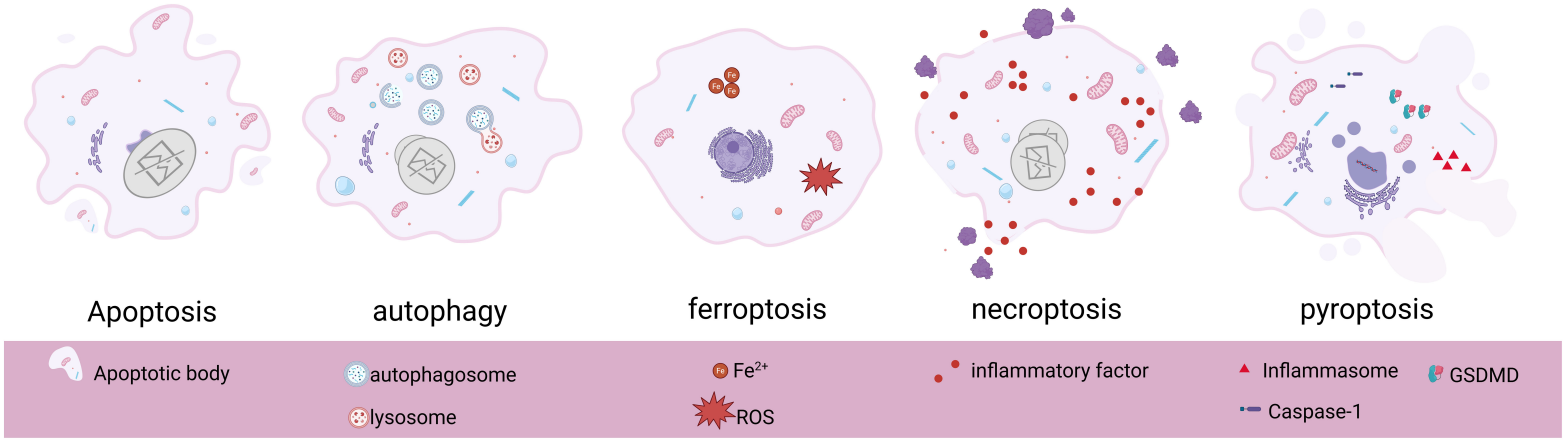
Basic morphological features and pathological changes of apoptosis, autophagy, ferroptosis, necroptosis, and pyroptosis. Created in BioRender. Yang, X. (2025) https://BioRender.com/s86x403.

As an evolutionarily highly conserved catabolic process, the protective mechanism of autophagy is unique to biogenesis (26). Under specific circumstances such as ischemia and hypoxia, nutrient deficiency, aging, or injury in cells, autophagy acts as a self-protective mechanism to achieve self-degradation and removal of damaged substances from the cells (27), which plays an important role in maintaining cellular homeostasis and survival, and this is what makes autophagy unique from other modes of programmed cell death. The different biogenesis and morphological alterations of several programmed cell deaths are shown below (**Table 1**, **Figure 1**).

**Table 1:** Biosignature and induction parameter of autophagy, ferroptosis, apoptosis, necroptosis, and pyroptosis.

Biosignature and induction parameter	autophagy	ferroptosis	apoptosis	necroptosis	pyroptosis
Morphological features	Cell membrane structural integrity, volume reduction, altered nuclear spacing, cytoplasmic vacuolization, and autophagic lysosome formation (28, 29)	Cell swelling, rupture of the plasma membrane, reduction in mitochondrial volume, reduction in mitochondrial cristae (30, 31), and no significant morphological changes in the nucleus	DNA fragmentation, cell shrinkage, chromatin and cytoplasm condensation, nucleolus rupture, plasma membrane blistering, apoptotic vesicle formation (32)	Swelling of cells and organelles, explosive rupture of the plasma membrane, infiltration or activation of a large number of inflammatory cells (33, 34), and no obvious morphologic changes in intranuclear chromatin (35)	DNA damage and nuclear condensation (36), the cell membrane is damaged to form perforations, little cell swelling, membrane rupture, cytoplasm flattening, and release of cell contents (37, 38).
Biochemical characteristics	Conversion of LC3-I to LC3-II, catabolism of autophagy substrates (e.g., p62) (39), increased lysosomal activation (40)	Oxidative damage to nuclear DNA, decreased GSH and GPX4 expression, and increased divalent iron accumulation and lipid peroxidation (41)	caspase activation and kinase cleavage (42)	RIP1, RIP3, and MLKL activation and reduced ATP levels, Caspase-independent, but inhibited by caspase-8-mediated cleavage of RIPK1 (43)	Caspase1/3/4/5/8/11 activation mediates GSDMD protein family cleavage, Activation of inflammatory vesicles, and release of large amounts of inflammatory factors (44–46)
biomarkers	LC3, p62, Beclin-1, ATG	ROS, GPX4, GSH, MDA, Fe^2+^	Caspase, Bcl-2, Bax, Ca^2+^	RIPK1, RIPK3, MLKL	Caspase-1, GSDMD, NLRP3, IL-1β
contributing factor	Intra- and extracellular stimuli such as glucose deprivation, hypoxia, nutrient deficiency, caloric restriction, oxidative stress, DNA damage, cytotoxic agents, and growth factor deficiency (47–49)	Lipid peroxidation and iron accumulation (41)	Nutrient starvation, Mechanical load, Abnormal metabolic conditions, disruption to the Circadian rhythms, bacterial infections, and virus infection (50)	Inflammatory or pathogenic infections (51)	Microbial (bacterial, fungal, viral) infections, chemical factors (52)

## 3 Autophagy mechanisms

ULK1, the mammalian homolog of the yeast Atg1 kinase, is a key kinase in the initiation of autophagy and nucleation process (53), and interacts with ATG13, FIP200/Atg17, Atg29, and Atg31 to form the ULK1/Atg1 complex, which regulates autophagy in an mTOR-dependent manner (54). Under sufficient nutrition, mTOR C1 is activated to inhibit autophagy by phosphorylating ULK1 and ATG13. Under nutrient limitation, AMPK first responds to hunger signals (55) and inhibits mTOR C1 on the surface of lysosomes, leading to rapid dephosphorylation of ULK1 and ATG13 and activation of ULK1 kinase (56), inducing the formation of autophagy precursor structures (also known as phagophore assembly site complexes, PAS) (57).

The main function of PAS is to recruit autophagic proteins, which play a crucial role in the initiation of autophagy (58), and the ULK1/Atg1 kinase complex is essential for recruiting Atg proteins to PAS (59). Beclin-1, the mammalian cellular homolog of the yeast autophagy-related gene Atg6, is an important component of the autophagy initiation complex and a key regulator of nucleation and can be inhibited by Bcl-2 (60). It can recruit autophagy proteins by interacting with PI3K (phosphatidylinositol 3-kinase)-like signaling pathways and forms a PI3K complex with VPS34, VPS15, and Atg14/ATG14L that is recruited to the PAS (61). Recent studies have shown that the PI3K complex targeting the PAS is mediated by the Atg1/ULK1 complex, ATG9, Vac8 (62), subsequently, the PI3K complex that is recruited to the PAS interacts with and binds to ATG13 in the ULK1 complex to participate in phagocytosis vesicle (as known as “detached membrane”) formation (63).

Activation of the PI3K complex leads to localized enrichment of PI3P (64), and the WIPI3/4 proteins sense the presence of PI3P and subsequently recruit the lipid transfer enzyme ATG2 into growing phagocytic vesicles, which subsequently mediates the transfer of lipids to the outer membranes of autophagic vesicles to achieve segregated membrane amplification and gradual segregation of cargoes, promoting autophagosome closure and maturation (65, 66). In addition to ATG2, WIPI1–4 also recruits the ATG5-ATG12-ATG16 complex, which mediates the coupling of LC3 to PE (67) and contributes to the correct assembly of autophagosomes (68). ATG9 is the only transmembrane protein identified in the core macroautophagy machinery (69) and is required for the formation of isolated compartments called autophagosomes (70). Most ATG9 is localized to small vesicles of Golgi origin (71), after autophagy induction, ATG9 is recruited to the PAS in response to Atg17, which is phosphorylated as a direct target of ULK1/Atg1 kinase, and phosphorylated Atg9 also recruits Atg8 and Atg18 to the PAS, which is required for the subsequent elongation of the separation membrane (72). Later studies showed that PAS localization of ATG9 vesicles is also achieved by binding to the HORMA structural domain of Atg13 (73). Notably, ATG9 circulates between the PAS and peripheral reservoirs (74, 75), is involved in the delivery of membranes required for the elongation and closure of separating membranes (58), and is important for autophagic processes. In addition, this cycling process is regulated by Atg18 and Atg2, independent of the Atg1 complex (76). Moreover, the cyclic transport of ATG9 vesicles balances ATG2-mediated lipid transfer, and the two autophagy-associated proteins coordinate to promote autophagosome growth and maturation (77).

In addition, the formation of autophagosomes possessing a bilayer membrane structure cannot be separated from the mediation of two ubiquitin-like coupling systems, ATG12 and LC3/Atg8. As mentioned previously, the ATG12-ATG5- ATG16 complex can mediate the coupling of LC3 to PE and promote the correct assembly of autophagosomes, moreover, it can stabilize PAS by interacting with the Atg1 complex (78). LC3 is widely used as a marker for the detection of autophagy and also serves as a docking site for a growing number of autophagy cargo receptors, LC3 can covalently couple to the membrane lipid phosphatidylinositol PE through a series of protein-lipid cascade reactions, a modification known as LC3 lipoylation, which allows LC3 to attach to autophagic vesicles, not only helping to recognize the vesicles as autophagic vesicles but also contributing to vesicle amplification and recruitment of cargo proteins or other autophagy proteins (79, 80). ATG7 and ATG3 play important roles in LC3 lipidation modification, LC3 I is activated by ATG7 and transferred to Atg3 to bind to PE and be modified to LC3 II, which is subsequently recruited to autophagic vesicle membranes. Notably, the lipidation of LC3 is blocked when ATG7 is deleted, which leads to limited expansion of the phagophore membrane, cells show “autophagy deficiency”, however, the molecular mechanism by which ATG7 activation occurs remains unclear (81). Subsequently, autophagic vesicle membranes continue to elongate and close to form autophagosomes, after which LC3 interacts with a variety of kinesins and kinetics proteins to facilitate autophagosome translocation to the lysosome for fusion (82), which leads to the formation of autophagolysosomal complexes, ultimately, LC3II within the autophagosome is hydrolyzed by lysosomal protein hydrolases, whereas LC3II on the cytoplasmic surface of the autophagosome continues to be lipidated by Atg4 (83), amino acids and other small molecules that are produced by autophagic degradation are transported back to the cytoplasm for recycling or energy generation (84).

**Figure 2:**
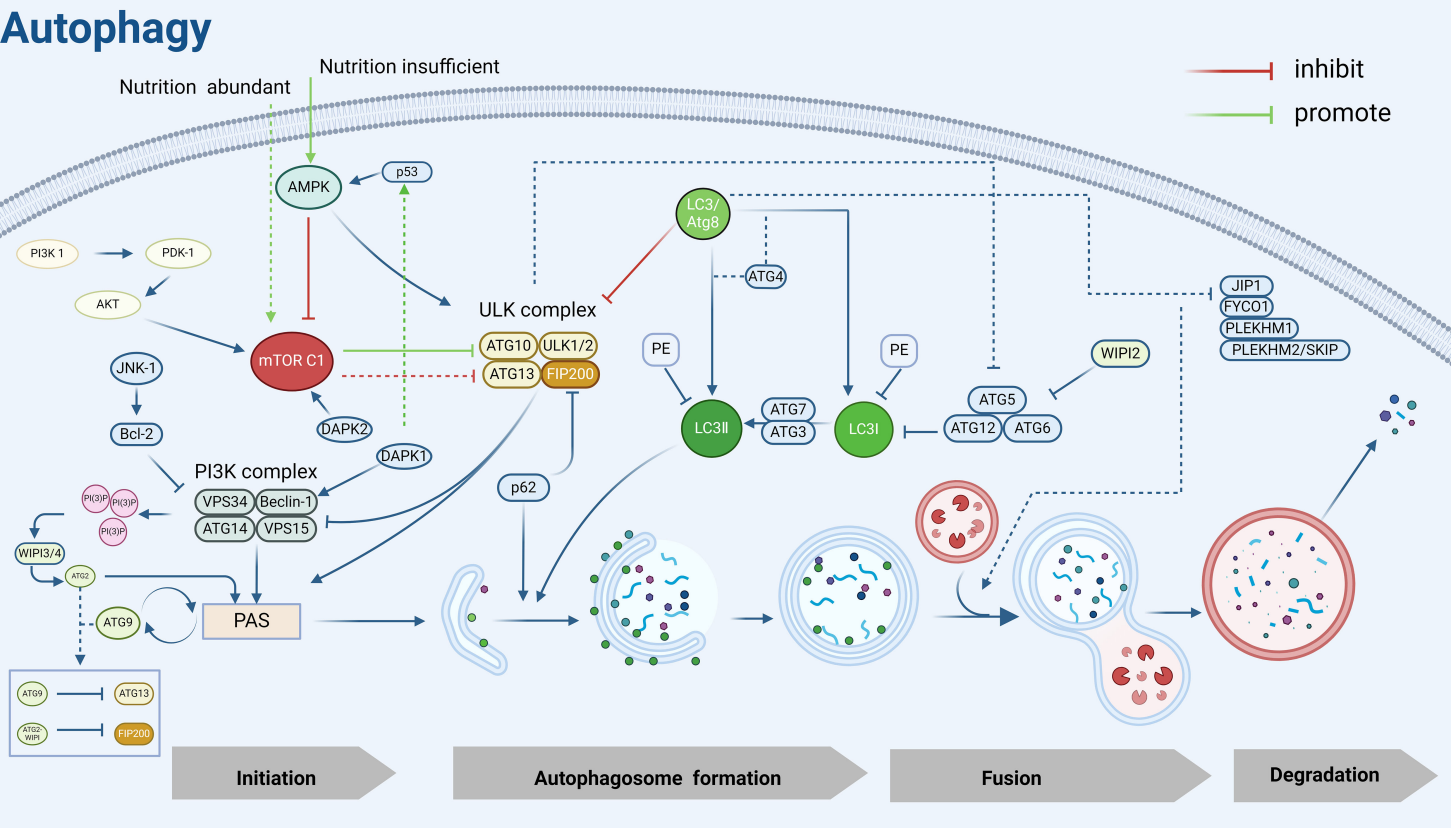
Autophagy mechanism diagram. PI3K, Phosphatidylinositol 3-kinase; PDK-1, 3-Phosphoinositide-dependent protein kinase 1; AKT, Protein kinase B; mTOR C1, Mechanistic Target of Rapamycin Complex 1; AMPK, Adenosine 5’-monophosphate (AMP)-activated protein kinase; JNK-1, c-Jun N-terminal kinase 1; Bcl-2, B-cell lymphoma-2; ULK, UNC51-like kinase; FIP200, FAK family kinase-interacting protein of 200 kDa; VPS34, Vacuolar protein sorting 34; VPS15, Vacuolar protein sorting 15; WIPI2/3/4, WD Repeat Domain, Phosphoinositide Interacting 2/3/4; PAS, Pre-autophagosomal structure; LC3, Microtubule-associated protein 1 light chain 3; PE, Phosphatidylethanolamine; p53, Tumor protein 53; p62, sequestosome 1(SQSTM1); DAPK2, Death-associated protein kinase 2; DAPK1, Death-associated protein kinase 1; ATG, Autophagy related gene; JIP1, JNK interacting protein 1; FYCO1, FYVE And Coiled-Coil Domain Autophagy Adaptor 1; PLEKHM1, Pleckstrin Homology And RUN Domain Containing M1; PLEKHM2/SKIP, Pleckstrin Homology And RUN Domain Containing M2(also known as SKIP). Created in BioRender. Yang, X. (2025) https://BioRender.com/v58z146.

In addition, the autophagy process is also regulated by various kinases such as DAPK1 and DAPK2. The death-associated protein kinase DAPK1 phosphorylates Beclin-1, which can promote autophagy both by weakening the interaction of Beclin-1 with Bcl-2 (85) and activating the p53 protein system, which affects the tendency of cellular autophagy (86). DAPK2 acts as a negative regulator of mTOR to modulate autophagy levels (87). P62, as the most important substrate protein for selective autophagy, promotes the recruitment of the ULK1 complex to initiate autophagosome formation by interacting with FIP200 (88) and can be doped for degradation in autophagosomes by interacting directly with LC3 on the separator membrane (89). The interaction of P62 with LC3 facilitates the maintenance of LC3 lipidation-modified separator membrane formation and provides a platform for autophagosome biogenesis, and impaired autophagy is accompanied by the accumulation of p62 (90)(**Figure 2**).

Because there are excellent articles in specialized fields dedicated to the mechanism of autophagy development, we made the above brief review of the complete autophagy process to elucidate the basic process and mechanism of autophagy development, to facilitate the scholars’ understanding of the autophagy-related mechanisms in the subsequent POI, and focused on the autophagy mechanism of POI and the study of non-hormone replacement targeted therapeutic strategies.

## 4 Autophagy mechanism in POI and molecular level-based intervention regulation

### 4.1 Epg5

The Ras gene from rat brain (RAB) GTPase is a class of regulatory proteins essential for eukaryotic endosomal membrane trafficking (91). Its member, Rab7, is located on late autophagosomes and lysosomes and is important for autophagosome maturation, autophagosome-lysosome fusion, and subsequent degradation of autophagosome contents (92). Ectopic P-granules autophagy protein 5 homolog (Epg5) is an effector of Rab7 that is recruited to autophagosomes/lysosomes during late autophagy by interacting with Rab7 and binds to LC3 to promote the fusion of autophagosomes and lysosomes (93). Epg5 deficiency leads to impaired autophagic flux and disrupts cellular homeostasis (94). In ovarian granulosa cells (GCs), sufficient autophagy ensures that appropriate levels of Wilms tumor 1 (WT1) protein are maintained to promote GC differentiation, whereas in the presence of insufficient autophagy, overaccumulation of WT1 inhibits the transcription and activation of the estrogen synthase CYP19A1 gene and the follicle-stimulating hormone receptor FSHR, resulting in defective GC differentiation and impaired E_2_ synthesis, which inhibit GC proliferation and induce cell death (95). However, the selective autophagic degradation of WT1 in GCs is mediated by Epg5, and knockout of Epg5 blocks autophagic flux and exhibits a POI-like phenotype in female mice (96). Therefore, the excessive accumulation of WT1 caused by defective autophagy of EPG5 in GCs is an intrinsic mechanism that causes POI.

### 4.2 NRF2 and FTO

Autophagy and the NRF2-Keap1 pathway crosstalk through the binding of the autophagy junction protein, p62, to the specific structural domains of both Keap1 (97), which is manifested as a defect in autophagy causes competitive inhibition of the NRF2-Keap1 interaction, resulting in NRF2 stabilization followed by transcriptional activation of NRF2 target genes (98). However, POI-related basic experiments have suggested that changes in NRF2 similarly affect cellular autophagy. In cisplatin-induced POI mice, NRF2 expression was upregulated, but this change was compensatory, and the initiation of “protective autophagy” was inadequate to resist the cumulative stress of cisplatin induction. Overexpression of NRF2 significantly increased the expression of autophagy-related proteins LC3, Beclin-1, and Bcl-2, as well as decreased the levels of autophagy substrate p62 and pro-apoptotic protein Bax, which increased the autophagy level of damaged granulosa cells and significantly enhanced the cellular resistance to apoptosis (99).

Fibroblast growth factor 2 (FGF2) is an antifibrotic factor that inhibits pro-fibrotic gene expression and suppresses fibroblast differentiation (100). It involved in reproductive processes such as follicle development (101–103), sex hormone regulation (104), granulosa cell proliferation, differentiation and apoptosis (102, 105), which plays a role in regulating ovarian function. Studies have shown that FGF2 plays a vital role in POI repair (105) and that FGF2 is also a key pathway for the induction of autophagy, its expression is upregulated to promote autophagy (106). Studies have shown that treatment of damaged granulosa cells with exogenous FGF2 increases the expression levels of NRF2 and LC3 in ovarian granulosa cells of POI mice, promotes NRF2 nuclear translocation, and enhances the anti-apoptotic capacity of damaged granulosa cells through activation of the autophagy pathway to play a reparative role (99)POI. In addition, FGF2 can activate autophagy and inhibit apoptosis in damaged granulosa cells by promoting the expression of Fat mass and obesity-associated protein (FTO). FTO contributes to the development and maturation of oocytes (107), and its expression is significantly reduced in ovarian tissues of POI patients and mice (108). Exogenous addition of FGF2 up-regulated the expression level of FTO in POI granulosa cells, promoted the accumulation of autophagic follicles, inhibited the apoptosis of granulosa cells, and enhanced the viability of cell proliferation (109). The above findings indicate that NRF2 and FTO are aberrantly expressed in POI and are associated with autophagy mechanisms, moreover, the expression levels of both NRF2 and FTO are regulated through the intervention of FGF2.

### 4.3 HSP70

Protein quality control is fundamental to the maintenance of cellular homeostasis, which depends on sustained protein degradation and resynthesis, the former is achieved through autophagy, the ubiquitin-proteasome system, and other lysosome-dependent degradation pathways, and the latter concerning the regulation of protein folding and repair by heat shock proteins (110). Autophagy and the heat shock response represent 2 functionally distinct but complementary systems of cellular protein quality control, and the heat shock response inhibits autophagy under conditions where both systems are activated (111). Heat shock protein HSP70 is the central hub of the protein homeostasis network, which protects cells from protein homeostasis disruption induced by oxidative stress, various pathological factors, and organismal aging (112, 113). During female reproduction, hsp70 leads to decreased autophagy and reduced oocyte viability, triggering a Premature ovarian insufficiency-like phenotype that affects fertility and pregnancy outcomes (114), high serum levels of HSP70 have been accepted to reflect ovarian damage and disease severity (115).

### 4.4 PI3K/AKT, MAPK and mTOR

The mammalian target of rapamycin (mTOR) is a core regulator of autophagy, regulated by different upstream signaling pathways (116), including the most important PI3K/AKT and MAPK/ERK signaling pathways (117). A review study showed that mTOR signaling plays an important role in female reproduction, such as follicular development, granulosa cell proliferation and ovarian aging, and that activation of the mTOR pathway protects the ovarian reserve and extends the life span of the ovary (118). PI3K/AKT/mTOR has negative feedback regulation of autophagy. Studies have shown that exposure to the organophosphate insecticide diazinon (DZN) leads to excessive ROS accumulation and DNA damage in porcine ovarian granulosa cells, which in turn induces apoptosis and autophagy through inhibition of the PI3K-AKT pathway and increases the risk of Premature ovarian insufficiency and follicular atresia (119). The results of many experimental articles suggest that targeting the PI3K/AKT/mTOR pathway by certain therapies can promote autophagy or inhibit excessive autophagy, thereby increasing the number of follicles at all levels, reducing follicular atresia, and alleviating ovarian aging (120–125).

Mitogen-activated protein kinase (MAPK) is a key driver of early autophagy initiation and autophagic vesicle assembly (126). There are multiple mammalian MAPK pathways, including extracellular regulated protein kinase 1/2(ERK1/2); c-Jun N-terminal kinase (JNK) also known as stress protein-activated kinase (SAPK); and p38 MAPK (127).

The above MAPK pathways are linked to autophagy to different degrees, and their functions exhibit differences. JNK not only regulates ATF-2 and p53 transcription factors, but also phosphorylates cytoskeletal proteins, and mitochondria-associated proteins such as Bcl-2 (128, 129), which regulates cell proliferation, apoptosis, and DNA damage repair (130), and activates autophagy by disrupting the Bcl-2/Beclin-1 complex (131). Meanwhile, activation of p38 MAPK induces ULK1 phosphorylation and disrupts the ULK1-Atg13 complex, thereby inhibiting autophagy (132).

ERK regulates the expression of multiple nuclear transcription factors and proteins to participate in various biological responses such as oxidative stress, apoptosis, and autophagy and affects altered mitochondrial dynamics; moreover, inhibition of ERK impairs mitochondrial activity and disrupts intracellular metabolic processes, which enhances dependence on autophagy and increases autophagic flux (133). Distinct from MAPK, adenosine monophosphate-activated protein kinase (AMPK) is a key energy sensor that regulates cellular metabolism to maintain energetic homeostasis, and promotes autophagy by activating the mammalian autophagy initiation kinase ULK1 under cellular energy and nutrient deficiency. However, this effect is disrupted by highly active mTOR under nutrient-rich conditions to inhibit the ULK1 activation and the start of autophagy (134). It has been shown that AMPK/mTOR is an important autophagic antioxidant pathway capable of attenuating oxidative stress damage in female reproduction and plays an important role in alleviating oocyte senescence (135).

In the studies of pharmacological interventions in POI cited later in this article, all of the above pathways have been shown to play an important role in regulating autophagy in POI and ameliorating ovarian senescence.

### 4.5 Mcl-1

Myeloid cell leukemia-1 (Mcl-1) is a unique antiapoptotic Bcl-2 member that is critical for mitochondrial homeostasis (136) and plays a key role in the control of survival and death of a wide range of cells (137). Previous studies have shown that Mcl-1 is not only involved in apoptotic mechanisms but is also involved in mitochondrial quality control via autophagy (138), and is an important molecular bridge between autophagy and apoptosis. Since the current studies are contradictory, Mcl-1 deletion in diverse tissues and organs exerts distinct impacts on autophagy. Therefore, its function in regulating autophagy is unclear as current studies are conflicting (136).

OMARI et al. evaluated the expression of Mcl-1 in mouse oocytes at different developmental stages, and the results showed that the expression of the Mcl-1 transcription factor decreased significantly with age, suggesting that Mcl-1 may be an important regulator of oocyte survival. To further investigate the role of Mcl-1 in oocytes, the researchers specifically excised Mcl-1 from mouse oocytes. The results showed that Mcl-1 defects led to the development of reduced oocyte numbers at all levels, increased follicular atresia, and oocyte depletion, which ultimately led to the ovarian premature aging-like phenotype of mice with impaired ovulation and reduced fertility, and the results at the molecular level showed that the pro-apoptotic factor Bax elevation, increased Beclin-1, LC3, and lysosome-associated membrane protein 2 (LAMP-2), suggesting that Mcl-1 deficiency promotes apoptosis to accelerate oocyte death at all levels to induce Premature ovarian insufficiency, while at the same time being able to activate cellular autophagy in response to mitochondrial dysfunction (139).

### 4.6 Tet

Tet enzyme is a DNA demethylase that plays an important role in DNA demethylation during meiosis in primordial germ cells and oocytes, as well as in the pluripotent differentiation of embryonic stem cells (140, 141). Tet1 deficiency increases organelle fission in oocytes, which is associated with defective ubiquitination and reduced autophagy, and is detrimental to the removal of damaged or senescent organelles from the cell, leading to a decline in oocyte quality, as well as in oocyte number and follicular reserve, contributing to POI. Moreover, Tet1 accelerates reproductive failure with age (142). However, the exact regulatory mechanism of Tet1 on POI autophagy is yet to be elucidated. Studies have shown that Tet1 mediates the methylation of autophagy promoter regions (143). Thus it is hypothesized that Tet1 affects autophagy in POI oocytes by altering the methylation levels of autophagy-related genes, and the specific molecular mechanisms remain to be further investigated.

### 4.7 TRIM28

The tripartite motif-containing protein superfamily 28 (TRIM28) is an autophagy regulator, which can inhibit autophagy by promoting the proteasomal degradation of AMPK (144). The level of TRIM28 protein in the ovarian GCs of POI mice was reduced by OS, leading to an increase of autophagy marker proteins ATG5 and LC3B-II, as well as the down-regulation of P62, which triggered the abnormal excessive autophagy. Overexpression of TRIM28 significantly improved the changes of the above indexes, inhibited autophagy in GCs, and increased the levels of P16, HO-1 and SOD2, which alleviated OS and aging, improved hormone levels and restored the ovarian reserve in POI mice (145).

### 4.8 LncRNA

LncRNAs are key regulators in the development of various human diseases, including reproductive disorders, and regulate the normal development of GC, follicle, and ovary by mediating multiple mechanisms (146), and their aberrant transcription is closely associated with the occurrence and development of POI.

In studies of Premature ovarian insufficiency, lncRNA nuclear-enriched abundant transcript 1 (NEAT1) and STC2 are downregulated in POI mice (147, 148), whereas miR-654 is upregulated in the plasma of POI patients (149). Bioinformatics software found a direct relationship between miR-654 and NEAT1, and the literature reported that STC2 is one of the targets of miR-654. By overexpressing NEAT1 can reduce the expression of miR-654, and regulate the STC2/MAPK pathway, inhibited cyclophosphamide-induced apoptosis and excessive autophagy in POI granulosa cells (150), and repaired POI ovarian damage. In addition, among cisplatin-induced POI granulosa cells, lncRNA HOTAIR was downregulated, leading to upregulation of miR-148b-3p, downregulation of ATG14, and inhibition of autophagy. Overexpression of HOTAIR not only improved the expression levels of miR-148b-3p and ATG14, but also upregulated the levels of ATG5, Beclin1, and SIRT1, and downregulated P62/SQSTM1, promoting autophagy and alleviating ovarian aging (151).

### 4.9 miRNA

MicroRNAs are major upstream regulators of the autophagy pathway, regulating autophagy by targeting autophagy-related genes or autophagy complexes at different stages of autophagy induction, autophagic vesicle nucleation, and vesicle elongation and closure (152), for example, MicroRNAs can directly target autophagy key proteins mTOR, ULK1/2, BECN1/Beclin-1, etc. to regulate their activities negatively (153, 154). Notably, microRNAs are the most abundant class of microRNAs in the ovary and play an important role in regulating ovarian function (155). Existing studies have demonstrated the potential of miR-644-5p, miR-21, miR-144-5p, and miR-146b-5p in the treatment of POI and restoration of ovarian function (156–159). In addition, miR-379, miR-15b, miR-691, miR-872, and miR-1897-5p have been reported as potentially useful markers of ovarian dysfunction (160). Extracellular vesicles of embryonic stem cell origin rejuvenate senescent cells both *in vivo* and *in vitro*, and the highly enriched miRNA-15b-5p within them are potent activators that mediate the rejuvenation of senescent cells (161). The anti-aging gene Klotho is closely related to the hypothalamic-pituitary-ovarian axis and plays a key role in the development of reproductive diseases, participating in the regulation of fibroblast growth factor-Klotho endocrine system dysfunction, the accumulation of oxidative stress, and the inhibition of autophagy, which ultimately affects follicular ontogeny, development, ovulation, or atresia (162). In cyclophosphamide-induced POI mouse granulosa cells, the expression level of miRNA-15b was elevated, and the expression of the anti-aging gene α-Klotho was significantly reduced. MiRNA-15b reduces the oxidative stress-related expression factors Superoxide Dismutase (SOD) and ATP levels by inhibiting the expression of α-Klotho in ovarian granulosa cells, causing ROS accumulation, the expression level of autophagy-related proteins was reduced, and autophagy activity and ROS scavenging ability were weakened (163).

In addition, in the cyclophosphamide-induced POI cell model, miR-497-3p was up-regulated, which inhibited Klotho transcription by targeting KLF4, and ultimately led to the inactivation of the PI3K/AKT/mTOR pathway, which promoted DNA damage, activated autophagy and apoptosis, and accelerated follicle depletion to trigger ovarian senescence in POI cells, whereas knocking down miR-497-3p reversed the damage and delayed ovarian aging (164). And in 4-Vinylcyclohexene Diepoxide (VCD)-induced POI rats and granulosa cell models, miR-144 was down-regulated, and overexpression of miR-144 increased AKT/mTOR phosphorylation levels, inhibited excessive autophagy, and alleviated ovarian damage (165)(**Table 2**).

**Table 2:** Autophagy mechanisms in POI based on molecular level.

Classification	Expression of genes or protein pathways	Downstream mechanisms	Conclusion	Intervention regulation	Effectiveness of the intervention	Research target	Bibliography
Gene or protein pathway	Epg5↓	WT1↑	inhibition of autophagic flux, defective GC differentiation, decreased proliferative capacity, impaired E2 synthesis, leading to POI	——	——	POI mice	(96)
	NRF2↑	——	Initiates protective autophagy	Overexpressing NRF2	LC3, Beclin-1, Bcl-2↑, p62, BAX↓, Compensatory increase of autophagy level and enhancement of anti-apoptotic capacity	POImice	(99)
				Exogenously added FGF2	NRF2↑, LC3↑, Activation of autophagy and enhancement of granulocyte resistance to apoptosis		
	FTO↓	——	——		FTO↑, BAX mRNA and protein↓, p62↓, Bcl-2, LC3 B, beclin1↑, Promotion of autophagic follicle accumulation and inhibition of granulocyte apoptosis	POI patients and mice	(109)
	HSP70↑	——	Inhibition of autophagy	——	——	——	(166)
	Mcl-1↓	BAX↑, caspase ↑, Beclin-1↑, LC3 A↑ , LAMP-2↑	Promotes apoptosis, activates autophagy, severely depletes oocytes at all levels, increases follicular atresia, and decreases oocyte viability	——	——	normal mice	(139)
	TRIM28↓	ATG5, LC3B-II↑, P62↓	Triggers autophagy and induces granulosa cell senescence, leading to follicular atresia and POI	Overexpressing TRIM28	ATG5, LC3B-II↓, P62, P16, HO-1 and SOD2↑, Inhibition of autophagy, OS and senescence in GCs, improvement of hormone levels, and restoration of ovarian reserve function in POI mice	POI mice	(145)
	PI3K/AKT/mTOR↓	ROS↑	DNA damage, promotion of autophagy or inhibition of excessive autophagy, induction of apoptosis, increased risk of premature ovarian failure and follicular atresia	——	——	Porcine ovarian granulosa cells, POI mice, POI rats, ovarian granulosa cell models	(119–125)
LncRNA	NEAT1↓	miR-654↑, STC2↓	——	Overexpressing NEAT1	miR-654↓, Activation of STC2/MAPK pathway, BAX↓, cleaved-caspase 3↓, LC3 B↓, LC3 II/LC3 I↓, Bcl-2↑, p62 ↑, Inhibition of cyclophosphamide-induced apoptosis and excessive autophagy in POI granule cells	POI mice, POI patients	(150)
	HOTAIR↓	miR-148b-3p↑, ATG14↓	Inhibition of autophagy	Overexpressing HOTAIR	miR-148b-3p↓, ATG14↑, ATG5, Beclin1, SIRT1↑,P62/SQSTM1↓	Cisplatin -induced POI granulosa cell model	(151)
miRNA	miR-15b↑	α-Klotho↓, SOD, ATP↓, ROS↑, Autophagy-related proteins↓	Decreased autophagic activity and ROS scavenging capacity	——	——	POI mice	(163)
	miR-497-3p↑	KLF4↑, Klotho↓, PI3K/AKT/mTOR↑	Promoting DNA damage and apoptosis in POI cells	Knockdown of miR-497-3p	Inhibition of DNA damage and apoptosis in POI cells	Cyclophosphamide-induced POI cell models	(164)
	miR-144↓	——	——	Overexpressing miR-144	PTEN↓, p-AKT/p-mTOR↑, Inhibition of excessive autophagy	4-vinylcyclohexene diepoxide(VCD) -induced POI rats and granulosa cell models	(165)

↑ indicates upward adjustment, ↓ indicates downward adjustment.

## 5 Autophagy-based natural product therapeutic strategies for POI

### 5.1 Natural products of plant origin

#### 5.1.1 Flavonoids

As a kidney tonic Chinese medicine, cuscuta chinensis is commonly used in the treatment of gynecological endocrine diseases, which can reduce free radical production, promote the production of superoxide dismutase, regulate immunity, and delay aging (167). Flavonoids contained in Cuscuta chinensis, which have the effect of delaying aging and improving microcirculation, exert multifaceted effects on the endocrine functions of the hypothalamic-pituitary-gonadal axis, such as regulating the level of ovarian sex hormones (168), protecting the frozen viability of male germ cells and the sexual function of aged male rats (169, 170), improving glycolipid metabolism (135), etc. Total flavonoids from Semen Cuscuta (TFSC) combined with low-intensity pulsed ultrasound (LIPUS) have important roles in improving reproductive endocrine function, anti-menopausal osteoporosis, and antioxidant activity (171, 172). In POI rats, TFSC was able to decrease p-PI3K/PI3K, p-AKT/AKT, and p-mTOR/mTOR, elevate the level of Bcl-2 protein, reduce the expression of Bax, cleaved-caspase 3, as well as the apoptosis rate of granulosa cells. It also promoted the expression of Beclin-1 and LC3II/LC3I in GC cells, decreased the expression level of p62, increased the number of autophagic vesicles in GC, and promoted cellular autophagy. Thus, it significantly improved the estrous cycle, ovarian index and hormone levels of POI rats increased the number of follicles at all levels, inhibited follicular atresia, decreased the detection rate of senescent cells, and alleviated oocyte developmental disorders and hormonal endocrine disorders associated with Premature ovarian insufficiency (125).

#### 5.1.2 Polyphenols

Curcumin is a hydrophobic polyphenol extracted from the rhizome of the ginger family with antioxidant, anti-inflammatory, and anti-apoptotic effects (173). Capable of protecting ovarian granulosa cells from oxidative stress damage and inhibiting apoptosis through AMPK/mTOR pathway-mediated autophagy, as well as improving sex hormone levels and promoting follicular development (174). Another study showed that curcumin inhibits oxidative stress through the Nrf2/HO-1 and PI3K/AKT signaling pathways, exerts anti-apoptotic effects, increases follicle number, and attenuates the senescence phenotype, rescues ovarian damage induced by D-galactose in POI mice (175). As mentioned earlier, Nrf2/HO-1 and PI3K/AKT signaling pathways are related to autophagy regulation, but this study did not detect autophagy-related indexes or illustrate whether curcumin affects the changes of cellular autophagy levels through these two signaling pathways, which is doubtful and needs to be further verified. In addition, resveratrol is a polyphenolic natural compound that can act as an autophagy activator to regulate cell metabolism and differentiation (176). It can reduce selective autophagy cargo receptor-SQSTM1/p62, activate JAK2-p, increase LC3II/LC3I, promote granulocyte autophagy, and impede the immune mechanism of POI progression in mice, thus inhibiting POI and improving hormone levels (177).

#### 5.1.3 Imidazole heterocycles

Allantoin, a nitrogen-rich purine catabolic metabolite from yam, can ameliorate aging by scavenging ROS to alleviate oxidative stress in plants (178). As a natural phytoestrogen, allantoin has a positive effect on ovarian follicle development (179). Studies have shown that the overprotective effects of allantoin are related to apoptosis, autophagy, and pyroptosis. In cyclophosphamide-induced POI rats, allantoin down-regulated the expression of LC3 II/LC3 I, caspase-1, as well as the pro-inflammatory cytokine interleukin L-1β, up-regulated the level of NLRP3 inflammatory vesicles, inhibited mitochondrial autophagy and cellular pyroptosis and up-regulated the expression of Bcl-2. Reduced the apoptosis rate and ROS level of ovarian granulosa cells in POI rats, lowered the levels of FSH and LH, increased the level of E2, inhibited follicular atresia, increased the number of mature follicles, primordial follicles, and secondary follicles, and ameliorated ovarian injury in rats (180).

It has been previously reported that autophagy removes intracellular inflammatory vesicle components and cytokines such as NLRP3, reducing inflammatory vesicle activation and inflammatory responses and that autophagy dysfunction leads to NLRP3 inflammatory vesicle hyperactivation and hyperinflammation (181). In turn, inflammatory vesicles can modulate the autophagic process, initiating protective autophagy to inhibit excessive intracellular stress (182). Autophagy crosstalk with inflammatory responses regulated by NLRP3 inflammatory vesicles precedes. Therefore, it is hypothesized that the inhibitory effect of allantoin on cellular pyroptosis in this study may be achieved through autophagy regulation of NLRP3 inflammatory vesicle activity. However, the specific regulation mechanism of autophagy by allantoin is not clear, and the deeper regulation mechanism of POI autophagy by allantoin needs to be further investigated.

#### 5.1.4 Polysaccharides

Lycium barbarum polysaccharides (LBP) are the highest percentage of functional components in Chinese wolfberry (183), with anti-inflammatory, anti-apoptotic, and anti-aging effects (184). It has been revealed to play a role in the prevention and treatment of a variety of diseases (185–187). Notably, LBP improves oocyte quality and follicular status, repairs ovarian damage, and enhances ovarian reserve capacity in women (188). In POI, LBP can inhibit the accumulation of POI senescent cells and senescence-related secretory phenotypes (such as the pro-inflammatory cytokine IL-1β and the matrix-degrading enzymes MMP-1 and MMP-13, etc.), down-regulate the expression of the senescence marker factor p16 INK4a, and promote the activation of the AMPK/Sirt1 pathway, improve autophagy activity, increase the number of ovarian granulosa cells and follicles, alleviate aging and correct sex hormone disorders, and improve D-galactose-induced POI symptoms (189).

#### 5.1.5 Saponins

Curculigoside (CUR) with molecular formula C_22_H_26_O_11_ and molecular weight 466,448 is the main active ingredient in Curculigo orchoides, and exhibits pharmacological effects such as anti-inflammatory, antioxidant, and enhancing immune activity (190). CUR protects ovarian reserve function from CTX-induced injury by activating the AKT/mTOR signaling pathway, inhibiting excessive autophagy, and attenuating GC apoptosis. It is capable of elevating antioxidant enzymes and decreasing markers of redox reactions, reducing levels of autophagy markers LC3II, and increasing autophagy substrate p62. The therapeutic effect is mainly characterized by the reduction of atretic follicles, the rebalancing of sex hormone levels, and the development of endometrium and glands (122), unlike the previous promotion of autophagy to improve ovarian function. It is hypothesized that excessive autophagy exists in the ovarian cells of cyclophosphamide-induced POI mice, while CUR negative feedback regulates autophagic flux to protect ovarian function at this time.

Ginsenoside Rg1 belongs to the triterpenoid glycosides and is an abundant active ingredient in ginseng that exerts pharmacological effects in antioxidant and anti-aging and has been regarded as a potent phytoestrogen (191, 192) that exerts its estrogenic effects through the rapid activation of estrogen receptor signaling pathway without relying on ligand estrogenic effects (Q. G. 193). In reproductive disorders, Rg1 was able to improve fertility and reduce ovarian pathological damage in D-galactose (D-gal)-induced POI mice by enhancing anti-inflammatory and antioxidant capacities and reducing the expression of senescence signaling pathway proteins (194).

In POI rats, Rg1 was able to negatively regulate the PI3K/AKT/mTOR pathway, decrease the mRNA and protein expression of ovarian PI3K, Akt, and mTOR, and up-regulate the expression of LC3-II, leading to increased autophagy levels, as well as reduce the staining rate of positive ovarian β-galactosidase and the expression of the senescence marker p16 INK4a, thereby inhibiting the onset of ovarian senescence (121).

### 5.2 Natural products of marine biological origin

Pearl powder, derived from the marine organism pearl oyster, is widely used in biomedicine as a traditional Chinese medicine with antioxidant, anti-inflammatory, anti-aging, immunomodulatory, and wound-healing pharmacological effects (195). Studies have shown that pearl extracts have excellent antioxidant and anti-aging effects both *in vivo* and *in vitro*, and can be used to treat various aging diseases (196). Previous literature has indicated that pearl powder has good therapeutic effects on gynecological diseases such as early-onset ovarian insufficiency, menopausal syndrome, abnormal uterine bleeding, etc. It has also been revealed to have a good ovarian protective effect in POI disease, and this protective effect is mediated through the autophagy pathway. It was shown that 740 mg/kg of high-dose pearl powder could regulate hormone levels, improve the estrous cycle, and promote follicular development in rats. Further investigation of its regulatory mechanisms revealed that pearl powder significantly reduced the expression of cleaved caspase-3, Bax, and the MAPK transcription factors of ERK1/2, p38, and JNK, and increased the expression of autophagy proteins LC3II, Beclin-1 and p62, as well as the activities of antioxidant enzymes GSH, SOD, CAT, and GSH-PX, and lowered oxidative stress products ROS and MDA, which ultimately promoted autophagy, inhibited apoptosis, and attenuated oxidative stress in ovarian granulosa cells of POI rats to improve ovarian function (197)(**Table 3**).

**Table 3:** Autophagy-based natural product therapeutic strategies for POI.

Classification	Name	Structural formula and molecular formula	CAS number	Source	Research object	Intervention methods	Molecular mechanism	Treatment effect	References
Flavonoids	The total flavonoids from semen muscular (TFSC)	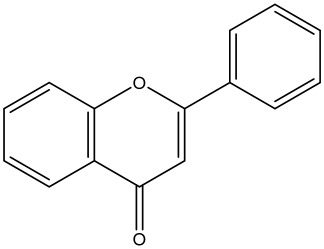 C_15_H_10_O_2_	525-82-6	Cuscuta chinensis	POI rats	140 mg·kg^−1^ TFSC by gavage every afternoon for 15 days.	p-PI3K/PI3K↓, p-AKT/AKT↓, p-mTOR/mTOR↓, Bcl-2 proteins↑, Bax↓, cleaved caspase 3/caspase 3↓, Beclin-1↑, LC3II/LC3I↑, p62↓	Inhibited granulosa cell apoptosis, promoted autophagy, significantly improved body weight, estrous cycle, ovarian index, uterine index, increased AMH, E2, P levels, decreased LH, FSH levels, increased primary and secondary follicle counts, decreased atretic follicle counts, and decreased detection of senescent cells in POI rats	(125)
polyphenols	curcumin	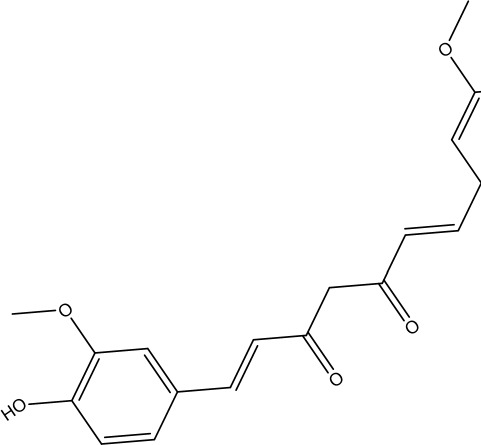 C_21_H_20_O_6_	458-37-7	ginger	POI mice	Oral curcumin treatment (50, 100, 200 mg·kg^−1^·d^−1^) given to POI mice.	AMPK/mTOR↓, BAX and caspase-3↓, LC3II↑, BECN1↑	Promoted autophagy, attenuated oxidative stress damage, inhibited apoptosis, improved sex hormone levels, and promoted follicular development.	(174)
	resveratrol	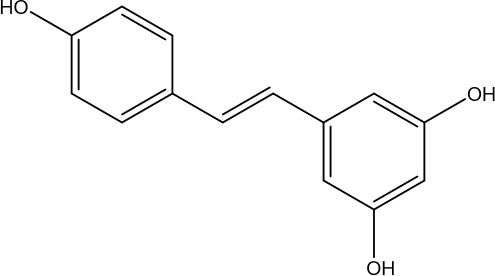 C_14_H_12_O_3_	501-36-0	Grapes, peanuts, Giant Knotweed Rhizome	POI mice	At a dosage of 50 mg/kg every other day via oral gavage, Lasting for 20 days	SQSTM1/p62↓, JAK2 -p↑, LC3II/LC3I↑	Promoted autophagy, increased E2 level, inhibited immune mechanisms affecting POI progression	(177).
Imidazole heterocycles	allantoin	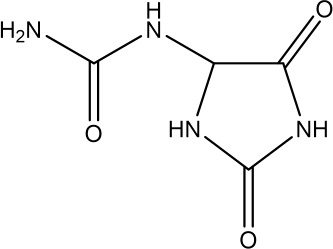 C_4_H_6_N_4_O_3_	97-59-6	dioscorea opposite Thunb	POI rats	ALL I 140 mg·kg^−1^ and ALL II 70 mg·kg^−1^ intraperitoneally once daily for 21 days. 140 mg·kg^−1^ was the optimal dose.	MMP↑, ROS↓, Bcl2↑, LC3II/LC3I↓, NLRP3↑, L-1β↓, caspase-1↓	Decreased FSH and LH hormone levels, increased E2 levels, decreased atretic follicles, increased mature follicles, primordial follicles, and secondary follicles, improved anorexia and estrous cycle in POI rats, inhibited granulosa cell apoptosis and mitochondrial autophagy, and blocked cellular pyroptosis	(180)
polysaccharide	Lycium barbarum polysaccharide (LBP)	NA	NA	wolfberry	POI mice	On the 15th day of the successful construction of the POI mouse model, LBP was administered by gavage at 60 mg·kg^−1^·d^−1^ for 28 days.	IL-1β↓, MMP-1↓, MMP-13↓, p16INK4a↓, p-AMPK/p-Sirt1↑	Promoted autophagy, increased the number of ovarian granulosa cells and follicles, alleviated aging, and corrected sex hormone disorders	(189)
saponins	Curculigoside (CUR)	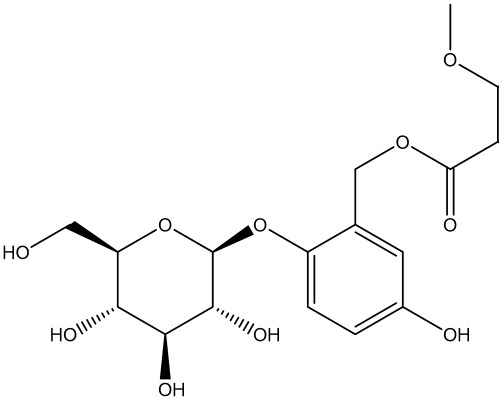 C_22_H_26_O_11_	85643-19-2	curculigo orchoides	POI mice	POI mice were given low, medium, and high doses of CUR by oral gavage (10 mg·kg^−1^·d^−1^, 20mg·kg^−1^·d^−1^and 40 mg·kg^−1^·d^−1^, respectively) for 28 days.	Activate AKT/mTOR, LC3II↓, p62↑	Inhibited excessive autophagy, attenuated GC apoptosis, reduced atretic follicles, restored sex hormone levels, promoted endometrial and glandular development, increased uterine integrity	(122)
	Ginsenoside	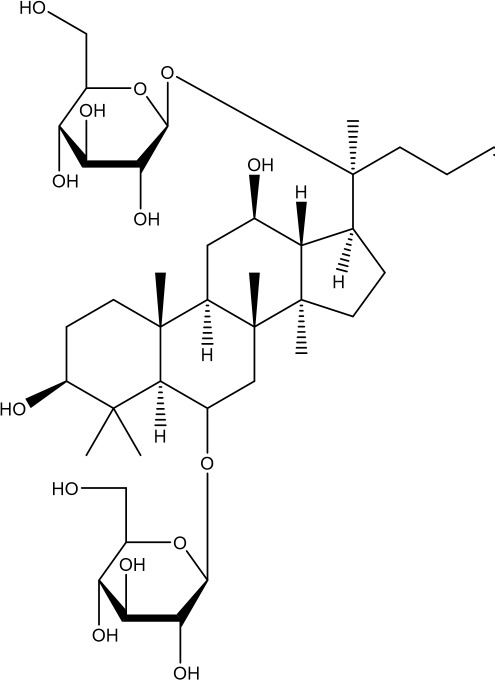 C_42_H_72_O_14_	22427-39-0	ginseng	POI mice	D-gal 200 mg-kg^-1^-d^-1^ was injected subcutaneously at the back of the neck for 42 days, and ginsenoside Rg120 mg·kg^−1^·d^−1^ was injected intraperitoneally starting on day 15 for 28 days.	PI3K, Akt, mTOR mRNA and protein↓, LC3II↑, P16INK4a↓	Inhibited PI3K/Akt/mTOR signaling pathway, activated autophagy, and inhibited ovarian aging.	(121)
Extracts from marine organisms	Pearl powder	NA	NA	pearl shell	POI rats	POI rats were given low, medium, and high doses of pearl extract by gavage (doses of 185 mg·kg^−1^·d^−1^, 370 mg·kg^−1^·d^−1,^ and 740 mg·kg^−1^·d^−1^, respectively) for 30 days.	Caspase-3↓, BAX↓, ERK1/2↓, MAPK transcription factors of p38 and JNK↓, LC3II↑, Beclin-1↑, p62↑, GSH↑, SOD↑, CAT↑, GSH-PX↑, ROS and MDA↓	Promotes autophagy, inhibits granulosa cell apoptosis, and mitigates ovarian damage by attenuating oxidative stress, elevating E2 and AMH hormone levels, and decreasing FSH and LH levels.	(197)

### 5.3 MSCs and their derivatives from different sources

Mesenchymal stem cells (MSCs) are pluripotent mesenchymal stromal cells derived from stromal tissues with plastic adhesion, self-renewal, and multispectral differentiation capabilities (198), which have shown great potential for research and clinical applications in the field of regenerative medicine (199). In addition, MSCs from a wide range of sources and can be extracted from various animal bone marrow, placenta, umbilical cord, amniotic fluid, menstrual blood, and adipose tissue, etc. MSCs from the above sources have been proven to be of great value in the treatment of POI (200), among which human umbilical cord MSCs (hUC-MSCs), with their advantages of being non-invasive, easy to obtain, and free from ethical controversy, have been more widely and intensively investigated in the treatment of POI, and have shown great potential for clinical application through further technological development and safety assessment in research (201). MSC therapy has become a promising therapeutic strategy for POI.

Autophagy can interact with signaling pathways that mediate the functional activity of various types of MSCs to improve the proliferation, differentiation activity, and paracrine effects of MSCs on other cells (202). The self-repairing function of MSCs and its influence on other cellular signaling pathways are closely related to the autophagy process. In addition, MSCs can promote tissue repair and regeneration and improve the microenvironment of damaged tissues by regulating autophagy (203). MSCs and autophagy interact and influence each other.

#### 5.3.1 Human umbilical cord mesenchymal stem cells and their secreted vesicles

HO-1 is an important antioxidant enzyme associated with autophagy, and both HO-1 and autophagy are recognized as part of the overall stress response, with both co-regulating to exert cytoprotective effects (204). Existing studies have shown that HO-1 was able to significantly restore impaired autophagic flux and lysosomal function and delay cellular senescence (99). In senescent mouse ovaries and oocytes, increased iron content and aberrant expression of iron metabolism proteins including HO-1 exhibit elevated lipid peroxidation and mitochondrial dysfunction, leading to localized redox state imbalance and decreased oocyte quality, and persistent iron death and mitochondrial autophagy (205). Yin, N et al. confirmed that the recovery of ovarian function was associated with the HO-1-mediated autophagy mechanism by comparing the results of HO-1/shHO-1 transfected hUC-MSC transplants. Elevated LC3 I/II, Atg5, and Beclin-1 mRNA and protein levels, and decreased p62 were observed in the HO-1 group, and in the shHO-1-MSC transplants of mice, the GCs’ autophagy was inhibited, as evidenced by reduced mRNA and protein levels of LC3 I/II and Beclin-1 and elevated p62. Further studies showed that HO-1 expressed in hUC-MSCs could help restore ovarian function in POI mice by activating autophagy mediated by the JNK/Bcl-2 signaling pathway (206).

Wenjie Dai et al. identified GCs as a regulatory target of MSCs in the improvement of ovarian function and found that hUC-MSCs could also activate the PI3K/AKT/mTOR pathway through the secretion of vascular endothelial growth factor A (VEGFA), which could attenuate oxidative stress and inhibit excessive autophagy in ovarian GCs, leading to an increase in sinus follicles and a decrease in atretic follicles, with a significant improvement in ovarian function and enhancement of fertility in POI rats (120).

In addition, 3D spheroidal hUC-MSCs can more accurately mimic the real microenvironment of cell differentiation, solve the cell adhesion problem (207), reduce tissue damage, promote angiogenesis, maintain their high survival rate and excellent paracrine ability in damaged tissues, and exhibit stronger antioxidant and anti-apoptotic abilities than 2D culture systems (208, 209). Moreover, this spherical MSC concentrated using certain methods can be preserved without refrigeration for easy transportation around the world for research and therapeutic applications (210). It has been shown that 3D spheroidal hUC-MSCs transplantation can improve POI more effectively than monolayer cultured hUC-MSCs and can reduce oxidative stress through paracrine function and prevent GC apoptosis and autophagy (208).

In human ovarian granulosa cells, excessive autophagy activates apoptosis, leading to cell death and accelerating follicle classification lar atresia, which reduces the number of follicles entering the growth pool, thus triggering POI (211), so inhibiting excessive autophagy in granulosa cells is also key to improving POI. Studies have shown that hUC-MSCs can attenuate excessive autophagy in GCs of POI mice, alleviate iron death and oxidative damage associated with excessive autophagy in granulosa cells, increase the number of follicles at all levels, improve the level of sex hormones, elevate the levels of proliferation-associated proteins PCNA and KI67, and slow down the aging of ovarian GCs (212). Moreover, extracellular vesicles secreted by human umbilical cord mesenchymal stem cells (hUC-MSCs-EVs) exhibit typical exosomal characteristics and have therapeutic effects on various degenerative diseases. Studies have shown that activation of the PI3K/AKT signaling pathway is the main mechanism by which hUC-MSCs-EVs protect the ovarian function in POI, which can reduce the level of cellular autophagy, significantly inhibit apoptosis in GCs of POI rats, improve ovarian morphology, promote follicular development and inhibit follicular atresia, as well as reduce the level of FSH, increase the level of E_2_ and AMH, and improve the reserve capacity of the ovary (124). Besides, hUC-MSCs-EVs were able to restore ovarian function in POI mice by up-regulating IGF-1, and hUC-MSCs-EVs carrying IGF-1 inhibited CTX-induced excessive autophagy and attenuated GC injury and ovarian dysfunction by activating the Hrf2/HO-1 pathway (213).

#### 5.3.2 Adipose-derived stem cells and their exosomes

Recent studies have shown that exosomes are closely related to intracellular autophagy in biogenesis and molecular signaling mechanisms (214). Adipose-derived stem cells exosome (ADSCs-Exo), an important intercellular messenger (215), has been identified as an important component of MSC paracrine secretion, and a growing number of studies have shown that ADSCs-Exo can be used as MSC alternative therapies for paracrine effects, including for the treatment of POI (200).

**Figure 3:**
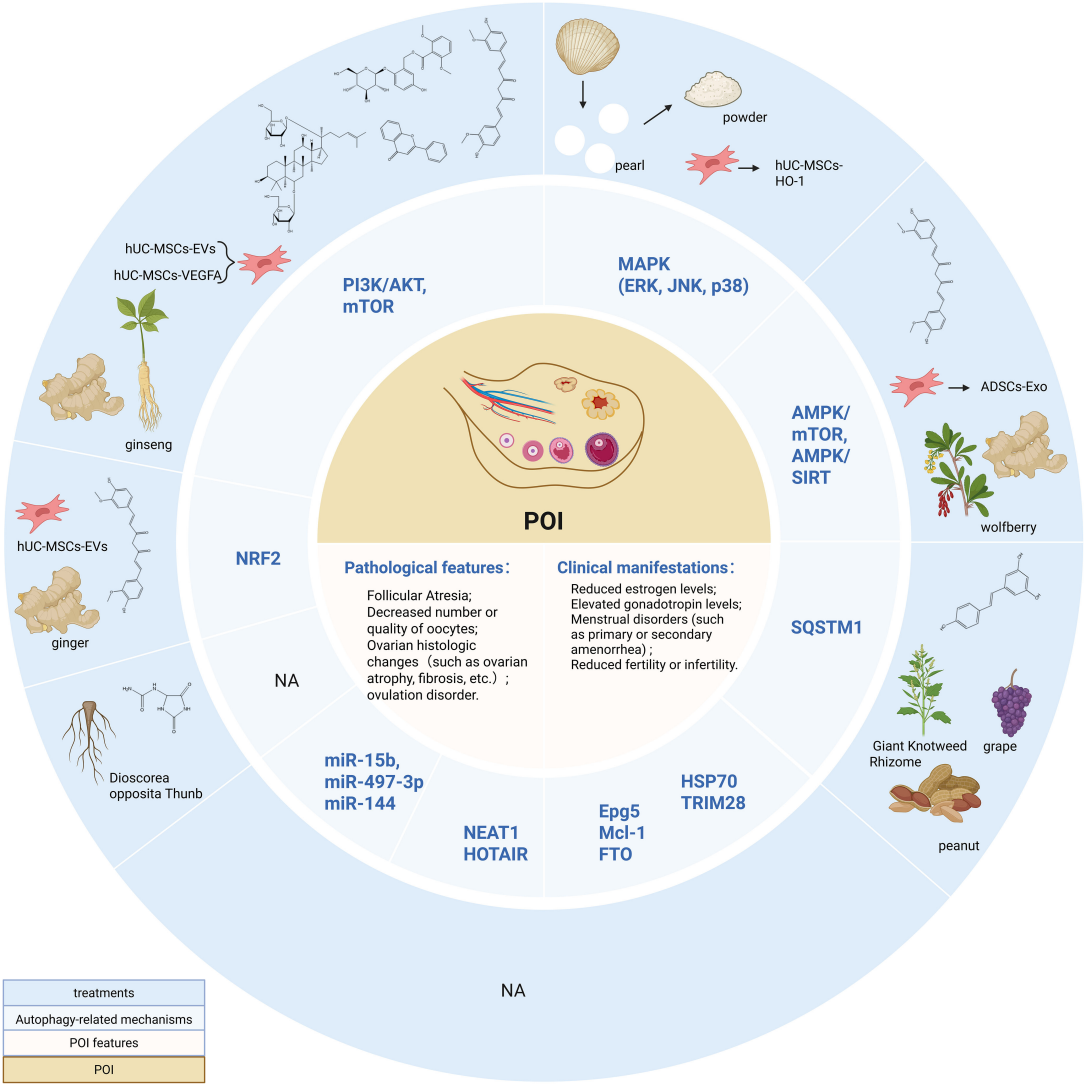
Autophagy-related Mechanisms and non-hormone Replacement Therapy Treatment in POI. PI3K/AKT, Phosphatidylinositol 3-kinase/Protein kinase B; mTOR, mammalian target of rapamycin; MAPK, Mitogen-activated protein kinase; ERK, extracellular regulated protein kinase; JNK, c-Jun N-terminal kinase; AMPK, adenosine monophosphate-activated protein kinase; SIRT, silent information regulator 1; Epg5, Ectopic P-granules autophagy protein 5 homologs; Mcl-1, Myeloid cell leukemia-1; HSP70, Heat shock protein 70; FTO, Fat mass and obesity-associated protein; NEAT1, nuclear-enriched abundant transcript 1; NRF2, Nuclear factor erythroid 2-related factor 2. Created in BioRender. Yang, X. (2025) https://BioRender.com/z84l329.

In POI mice, ADSCs-Exo reduced Beclin-1 and LC3II/LC3I protein levels and increased Bcl-2 expression by downregulating p-AMPK and upregulating p-mTOR, thereby inhibiting granulosa cell apoptosis and autophagy, attenuating pathological damage to ovarian tissues caused by oxidative stress, and improving ovarian function and morphology to counteract Premature ovarian insufficiency (123). Furthermore, a recent study demonstrated that hypo-Exos transferred miR-205-5p to enhance endothelial function and angiogenesis and alleviate POI by targeting the PTEN/PI3K/AKT/mTOR signaling pathway (216). This recent paper indicates that the treatment of POI by hypo-Exos is achieved through PI3K/AKT/mTOR-mediated angiogenesis. Although autophagy-related content is not mentioned, PI3K/AKT/mTOR is one of the most classical autophagy pathways known, and we believe that based on the results of this article there may be further studies to clarify the effect of hypo-Exos on the POI autophagy-related mechanism potential (**Table 4**, **Figure 3**).

**Table 4:** Stem cell therapeutic strategies based on POI autophagy mechanism.

Name	Source	Research object	Intervention methods	Molecular mechanism	Treatment effect	References
Umbilical cord mesenchymal stem cell HO-1	umbilical cord	POI mice	1 × 10^6^ 6th passaged MSCs were suspended using PBS and injected into POI mice.	HO-1↑, JNK/Bcl-2↑, LC3II/I, Atg5 and Beclin1 mRNA and protein↑, p62↓	Promoted autophagy and restored ovarian function	(206)
Umbilical cord mesenchymal stem cell VEGFA		POI rats	Tail vein injection of 1 × 10^6^/200µL MSCs	VEGFA, VEGFB, and PI3K/AKT/mTOR↑	Inhibited excessive autophagy in granulosa cells. Increased ovarian index and ovarian volume, increased the number of sinus follicles, decreased the number of atretic follicles, and improved estrus.	(120)
Umbilical cord mesenchymal stem cell		POI rats	3D cultured spherical hUC-MSCs (1×10^6^ cells) were injected and subsequent assays were performed after 48 days.	P62, Bcl-2 and PCNA↑, ATG5 and LC3II/I↓, CYP19A1, Bax, and Bcl-2↑	Reduced oxidative stress through paracrine effects, thereby inhibiting apoptosis and autophagy in GCs, improved POI more effectively than cells cultured in monolayers.	(208)
Umbilical cord mesenchymal stem cell		POI mice	10^6^/100 μL of hUC-MSCs suspension was injected into tail vein of mice, lasts for 28 days	NCOA4, FTH1, and LC3B↓, Fe^2+^↓, MDA↓, GSH↑	Attenuated excessive autophagy in granulosa cells and alleviated iron overload and oxidative damage associated with excessive autophagy, increased the number of follicles at all levels, decreased the level of FSH, increased the level of E2 and proliferation-associated proteins PCNA and KI67	(212)
Exosomes secreted by umbilical cord MSCs		POI rats, POI granule cell model	1×10^5^ mL^-1^ GC was injected into the plate, and 80 μg·mL^-1^ hUC-MSCs-EVs were added to the hUC-MSCs-EVs group and co-cultured with POI granulocytes, and POI rats were also treated with hUC-MSCs-EVs.	PI3K/Akt↑	Reduced the level of cellular autophagy, inhibited apoptosis of GCs in POI rats, improved ovarian morphology, promoted follicular development, inhibited follicular atresia, lowered the level of FSH, and increased the level of E2 and AMH hormones.	(124)
Extracellular vesicles secreted by umbilical cord MSCs		POI mice	POI mice were given 0.2 mL suspension encompassing 1 × 10^8^/mL hUC-MSCs-EVs through their tail veins once every two days for 6 consecutive weeks.	Nrf2/HO-1↑, ROS, MDA and Beclin-1↓, LC3↓, GSH↑	Inhibited excessive autophagy, attenuated CTX-induced GC damage and ovarian dysfunction, increased follicle number and serum E2 and AMH levels, decreased FSH and LH levels	(213)
Adipose-derived stem cell exosomes	fat	POI mice	POI mice were given tail vein injections of 100 μL ADSCs-Exo. , starting on the first day of modeling and injected every other day for 2 weeks.	p-AMPK↓, p-mTOR↑, Beclin-1↓, LC3II/LC3I↓, Bcl-2↑	Inhibited granulosa cell autophagy and apoptosis, attenuated pathological damage to ovarian tissues caused by oxidative stress in POI mice, and improved ovarian function and morphology.	(123)

## 6 Discussion

Long-term hormone replacement therapy (HRT) is currently the treatment of choice in the clinic, but recent studies have shown that populations receiving HRT are at higher risk for breast cancer (217), endometrial cancer (218), coronary heart disease (219), autoimmune disorders (220), as well as for undergoing a cholecystectomy (G. J. 221), suggesting that HRT produces a range of complications that are detrimental to women’s health, fails to fundamentally restore ovarian function, and has limited effect on improving patient fertility. Natural products and their compound components can inhibit aging by activating autophagy (222) and play a role in a variety of diseases such as Alzheimer’s disease (223), chronic kidney disease (224), and postmenopausal osteoporosis (225). MSCs and their secretions have also been extensively studied in a variety of diseases or pathologies such as acute kidney injury (226), peripheral nerve injury (227), testicular injury and fertility disorders (228), and promotion of skin wound healing (229). Autophagy is an important catabolic process of cellular self-cleansing responsible for the degradation of harmful and damaged intracellular substances, and autophagy has been shown to play a beneficial role in age-related processes and lifespan extension (224).

In recent years, stem cells and natural products have been found to play an important role in alleviating the clinical symptoms of POI and rescuing women’s fertility. In this paper, we explored how the different sources of natural products and stem cells can be used in the treatment of Premature ovarian insufficiency by regulating autophagy. We found that the current research on the molecular mechanism of POI autophagy involves the regulatory factors Epg5, NRF2, and Mcl-1, the transcriptional proteins FTO, HSP70, and Tet, the signaling pathways PI3K/AKT, MAPK, and mTOR, as well as the LncRNAs NEAT1 and miR-15b, miR479-3p. Notably, the aberrant expression of the above regulatory factors in POI other than NRF2 and Mcl-1 inhibits autophagy to varying degrees, leading to ovarian damage and inducing disease. Moreover, the up-regulation of NRF2 expression in POI mice was able to activate protective autophagy in ovarian tissues, but this was compensatory, and only by artificially intervening overexpression of NRF2 could autophagic flux be maximally increased, which could play a real protective role for the damaged ovaries in POI. Although knockdown of Mcl-1 was shown to activate autophagy, the main effect of knockdown was to promote apoptosis and accelerate oocyte depletion. It was hypothesized that the reduction of Mcl-1 in POI might be a slight protective autophagy initiated by cell injury, and this degree of autophagy also counteracts cell injury. In addition, PI3K/AKT/mTOR is a classical autophagy pathway, and the available findings suggest that it regulates autophagy comprehensively and deeply, both inhibiting autophagy and activating autophagic clearance mechanisms *in vivo*.

However, in general, the current development of non-hormonal replacement therapies targeting the autophagy mechanism of POI mentioned above is not deep and comprehensive enough. Total flavonoids of Cuscuta chinensis, imidazole heterocyclic compounds, polysaccharides, polyphenols, saponins, and pearl shell extracts from marine organisms are among the few active ingredients of natural products that have been investigated to the level of autophagy mechanism. In addition, different sources of MSCs and their exosome therapies are at the forefront of the literature to explore new therapeutic strategies for POI, among which hUC-MSCs are the most richly researched, and their secretion of HO-1, VEGFA, exosomes, and the innovative 3D sphere culture and transplantation technology have achieved remarkable results in the study of targeted autophagy for the treatment of POI.

An important problem in the research of POI natural product therapy is that there is less literature on natural product-targeted autophagy therapy for POI, and the research on the autophagy regulatory mechanism is limited and not extensive enough, in addition, the side effects and safety of natural products are relative, there are potential toxicological mechanisms of many active ingredients of natural products. In the study of POI autophagy, the evaluation of complications or adverse reactions caused by these natural products after treatment is insufficient, the research objects are limited to animals and cells, and the corresponding clinical studies have not been carried out yet. Therefore, multicenter, large-sample, double-blind, high-quality randomized controlled trials as well as scientific and cautious clinical trials are needed to accumulate more scientific evidence to support the safety and efficacy of natural product-targeted autophagy for POI. In addition, it is worth noting that the occurrence of the autophagy process in POI is often accompanied by crosstalk with other programmed cell death modalities or biogenesis processes, such as apoptosis, pyroptosis, mitochondrial autophagy, oxidative stress, and so on. In addition, other sources of natural products, such as terpenoids, glycosides, and other natural active ingredients isolated from algae, sponges, and mosses in the marine environment have antibacterial, antiviral, and antitumor effects (230), which have been demonstrated to be applied in clinical trial studies of neurodegenerative disorders (231) and breast cancer (232). Furthermore, with advances in microbiology, the effects of natural products from fungi on diseases of the female reproductive system have been extensively studied. Zearalenone, an estrogenic mycotoxin produced by Fusarium oxysporum, alters oocyte morphology, disrupts the estrous cycle, reduces hypothalamic-pituitary-ovarian axis activity, and disrupts female fertility (233), and the hyperthermophilic fungus, Aspergillus terraeus TM8, exerts its anticancer activity through apoptosis and has been implicated in the treatment of prostate cancer and breast cancer (234). Trace elements such as vitamins and minerals also play important roles in the female reproductive system. Cadmium and excess molybdenum can exacerbate ovarian damage by mediating necrotic apoptosis triggered by endoplasmic reticulum stress regulated by Th1/Th2 imbalance (235), while zinc and selenium improve follicular quality by improving mitochondrial dynamics and attenuating oxidative stress (236, 237). The above studies have demonstrated that various types of natural products have great potential to be explored in both scientific research and clinical practice of POI. Therefore, in the future, researchers should consider exploring more autophagy targets based on the crosstalk between autophagy and other processes, identifying and developing more diversified natural products with autophagy-regulating functions, and clarifying and evaluating their therapeutic ability for the treatment of POI, so as to promote their application in the treatment of POI.

Important issues identified in studies of MSC therapy for POI are that the use of MSCs for POI is still in the preclinical experimental stage based on concerns about the efficacy and safety of the current clinical application of MSCs in humans (201), and there are no definitive reports of clinical adverse effects, in addition, the significant variability in the different characteristics of different sources of MSCs in POI therapy has not yet been revealed. However, the efficacy of stem cell therapy has been demonstrated in many animal and cellular experiments, and the next step is to increase the research efforts, expand the sample size, and carry out cautious experiments in order to try to reveal the adverse effects and the advantages and disadvantages of stem cell therapy in POI treatment and to promote the clinical application of stem cell therapy. In addition, there is much evidence that MSCs from many different sources can treat POI by improving different pathological features, such as autologous menstrual blood-derived MSC transplantation improves ovarian function and restores the menstrual cycle in patients with POI, endometrial stem cells attenuate cisplatin-induced iron death of granulosa cells in POI by regulating the expression of Nrf2 (238), human embryonic-derived MSCs secreted VEGF, IGF-2, and HGF *in vitro*, which inhibited granulosa cell apoptosis, promoted angiogenesis and follicular growth, restored injured ovarian tissue structure and function, and salvaged the fertility of POI mice (239), and bone marrow-derived MSCs restored the fertility of POI mice through the up-regulation of FOXO1, GDF-9, and Fst genes and down-regulate TGF-β expression to promote granulosa cell proliferation and follicular development to treat cancer radiotherapy-induced POI (240). However, whether and how the above stem cell therapies treat POI by affecting autophagy has not been revealed, and more experiments are needed to enhance the exploration of the pathways linking MSCs from different sources and autophagy in POI in the future, to provide a molecular basis for stem cell transplantation and drug-targeted treatment of POI. Moreover, different MSCs from young and old, obese and non-obese populations showed differences in their ability to promote vascular endothelial cell formation, inhibit apoptosis, and enhance their proliferation, with senescent and obese-derived MSCs showing decreased protective and proliferative abilities, and obese population-derived MSCs exhibiting a senescence-associated secretory phenotype (241). Therefore, there are significant differences in the function, phenotype, and ethics of MSCs derived from different populations, and their therapeutic effects on POI are quite different, which provides new ideas for studying the therapeutic characteristics of MSCs derived from different populations.

## 7 Conclusion

In conclusion, future research on natural products and stem cell therapies in POI autophagy mechanism still faces great challenges, and more scientific trials need to be conducted to deeply investigate the mechanism of action, adverse effects, and clinical applications of these non-estrogenic replacement therapies in POI, to enhance the development and utilization of novel natural products in POI, and to accumulate more research evidence for retrospective analysis as well as for the further development of POI therapeutic strategies.
